# Paravertebral Block with Compound Betamethasone in Laparoscopic Cholecystectomy: A Double-blind Randomized Controlled Trial

**DOI:** 10.7759/cureus.6023

**Published:** 2019-10-29

**Authors:** Jinlei Li, Lili Li, Xiaoliang Zhang, Cong Li, Dong He, Jian Zhang, Chenxia Duan, Francisco Perese, Andrew Burzynski, Christopher L Wu, Feng Dai, Yun Xue

**Affiliations:** 1 Anesthesiology, Yale School of Medicine, New Haven, USA; 2 Pain, Fuling Central Hospital of Chongqing City, Chongqing, CHN; 3 Anesthesiology, Somnia Anesthesia, Memorial Medical Center, Las Cruces, USA; 4 Pain, Yale University, New Haven, USA; 5 Anesthesiology, Hospital for Special Surgery/Weill Cornell Medical College, New York, USA; 6 Biostatistics, Yale University, New Haven, USA; 7 Anesthesiology, Fuling Central Hospital of Chongqing City, Chongqing, CHN

**Keywords:** thoracic paravertebral, betamethasone, particulate glucocorticoid, laparoscopic cholecystectomy, block duration

## Abstract

Backgrounds

The aim of this study was to evaluate the utility of unilateral single injection thoracic paravertebral block (TPVB) with and without the addition of betamethasone for the acute pain management of patient’s undergoing laparoscopic cholecystectomy (LC).

Methods

Eligible patients were allocated randomly to three groups: (A) general anesthesia followed by surgeon infiltration at port sites with ropivacaine (n = 48), (B) general anesthesia after single injection TPVB at right T7-8 level with ropivacaine only, Ropi_TPVB (n = 43), and (C) general anesthesia after single injection TPVB with ropivacaine plus betamethasone, Ropi_Betamet_TPVB (n = 45). Primary outcome was TPVB duration assessed by the number of dermatomes at regular intervals up to 72 hours (h). Secondary outcomes included pain scores, analgesics consumption, and perioperative functional outcomes.

Results

The addition of betamethasone to ropivacaine in TPVB resulted in similar onset but significantly slower block regression between 4 h and 72 h as compared to ropivacaine alone (P < 0.001). When compared to the surgeon infiltration group, Ropi_TPVB and Ropi_Betamet_TPVB group had significantly lower pain scores for 24 h and 48 h, respectively, P ≤ 0.001. Both TPVB groups had less frequency of analgesics administration for 72 h, P < 0.001, and earlier mobilization, P < 0.001.

Conclusions

The addition of betamethasone to TPVB significantly prolonged block duration as compared to local anesthetic alone. TPVB both with and without the addition of betamethasone resulted in better perioperative analgesia and improved functional status when compared to surgical site local anesthetic infiltration.

## Introduction

Laparoscopic cholecystectomy (LC) is a common and high-volume surgical procedure worldwide. It was estimated that approximately 5.5 million cases were performed in non-federal community hospitals in the United States alone between 1998 and 2006 [1]. While considered a relatively less invasive alternative compared to open cholecystectomy, LC nevertheless constitutes a profound insult to patients with the duration of pain and post-operative functional recovery ranging from one to seven days. Regional anesthetic interventions are at the center of a myriad of acute pain management maneuvers. In particular, thoracic paravertebral blocks (TPVB) have proven to be as effective as the more invasive epidural anesthesia in treating somatic/visceral pain and promoting positive recovery metrics in abdominal procedures [2].

In this randomized, double-blind, controlled study of LC patients, we specifically investigated the effects of unilateral TPVB with or without adjuvants, combination of non-particulate and particulate betamethasone, versus surgeon infiltration at port sites, on blockade duration, pain control and other patient-centered perioperative outcomes. The regression of sensory block, an important factor for analgesia duration, was well studied in epidural anesthesia [3, 4], but nearly none was documented in paravertebral block. Our hypotheses were: (1) Perineural administration of betamethasone together with local anesthetic would prolong the duration of TPVB blockade; and (2) TPVB would provide superior post-operative analgesia as compared to surgeon infiltration. To our knowledge, this is the first randomized controlled trial (RCT) that investigates the prolongation effects of a mixture of non-particulate and particulate glucocorticoids such as betamethasone on TPVBs in the operating room and immediately postoperatively.

Clinical Trial Registration: This study was registered at http://www.chictr.org.cn, identifier ChiCTR1800016696.

## Materials and methods

Study design

This was a single center, prospective, randomized, double-blind, and controlled study with an institutional approval from the Ethic Committee of Fuling Central Hospital of Chongqing City in China. During the study period between April 12, 2017 and April 12, 2018, 283 LC patients were identified, 117 were excluded, 16 declined, therefore 150 patients were enrolled. Inclusion criteria consisted of: (1) age 18 to 75 years; (2) American Society of Anesthesiologists (ASA) status I-II; (3) elective LC; (4) weight >40 kg; (5) ability to self-administer opioid via PCA (standard of care in study hospital); and (6) overnight admission. Exclusion criteria included: (1) patient refusal, or patients with psychiatric or cognitive disorders; (2) a positive pregnancy test; (3) thoracic vertebral anomalies such as scoliosis or prior thoracic spine surgery; (4) coagulopathy; (5) systemic infection or local infection at planned injection sites; (6) allergy to any medications used in the study; (7) preexisting glucocorticoid use; (8) chronic pain; (9) diabetes mellitus (DM); (10) existing neuropathy. After an informed consent was obtained, 150 patients were divided into three groups per randomization table according to the pre-generalized random list in a 1:1:1 ratio: Local anesthetic Infiltration Analgesia Group (Control group), Ropivacaine single-injection TPVB Group (Ropi_TPVB), Ropivacaine & Betamethasone single-injection TPVB Group (Ropi_Betamet_TPVB).

Study protocol

1. *Intraoperative Study Protocol*

Upon entering the operating room, patients were monitored with ASA standard monitors, including SpO2, ECG, and non-invasive blood pressure. After infusion of 10 ml/kg lactate ringer was initiated, patients were given intravenous midazolam 0.04 mg/kg and sufentanil 0.1 mcg/kg.

All TPVBs were performed by the same investigator (LL), an experienced attending anesthesiologist. Under B mode-ultrasound guidance (Sonosite, S-Nerve), A 22-gauge Tuohy needle (B. Braun Medical Inc., Bethlehem, PA) was used to identify the right T7-8 paravertebral space under sterile condition in sitting position. A syringe filled with normal saline was connected to the needle. Upon identification of T7 transverse process, the needle was withdrawn slightly and re-directed caudally under the T7 transverse process. The needle was advanced until passing costotransverse ligament and 1 ml of normal saline was injected into paravertebral space. After visualization of intervertebral space expansion and pleural displacement away from the ultrasound probe under direct visualization, the patients in group Ropi_TPVB or Ropi_Betamet_TPVB would be injected with 0.5% ropivacaine hydrochloride (AstraZeneca) 14 ml + 0.9% normal saline 1 ml, or 0.5% ropivacaine hydrochloride 14 ml + 2.5 mg betamethasone dipropionate phosphate/1 mg betamethasone sodium phosphate in 1 ml (Merck Sharp & Dohme), respectively. The onset of block was assessed 15 minutes after block completion by evaluation of patient’s sensation to discern temperature change (with ice).

For all groups, an intravenous (IV) general anesthesia induction was then performed with intravenous propofol, sufentanil and rocuronium followed by endotracheal intubation.

Before surgical incision, patients in the control group were infiltrated with 0.5% ropivacaine 15 ml by the surgeon per standard protocols in this institute, with port I immediately to the right of the umbilicus, port II immediately below the xiphoid and 3 cm to the right of the midline, and port III 5 cm below the rib cage at right midclavicular line.

Anesthesia were subsequently maintained with a mixture of oxygen, air, and sevoflurane with end-tidal CO2 between 35 and 40 mmHg. Sufentanil 0.1 mcg/kg, up to 5 mcg, was injected for any increase in arterial blood pressure and/or heart rate >20% from baseline. All subjects received ondansetron 0.1 mg/kg, up to 4 mg, at the end of surgery.

2. *Postoperative Study Protocol*

All subsequent follow-ups were performed by the same investigator (XZ). Analgesic block levels and duration were assessed using patient’s temperature sensation change (with ice). The primary outcome was TPVB duration via the number of dermatomes affected on the ipsilateral side of TPVB placement, as a measure of duration of TPVB, assessed at 0.5 h, 2 h, 4 h, 8 h, 12 h, 24 h, 48 h and 72 h starting when patients left recovery room. Secondary outcomes included pain scores measured on a 0 to 10 numeric pain scale (NPS), the frequency and dosage of analgesics including oral and intravenous (IV) nonsteroidal anti-inflammatory agents (NSAIDs), and IV opioids up to 72 h at discharge. Surgical outcomes and quality metrics included preoperative and postoperative serum glucose level, first time ambulation, length of hospital stay, as well as any side effects such as dizziness, nausea, vomiting, drowsiness, hypotension, respiratory inhibition, intestinal paralysis/ileus, delayed wound healing, and wound infection were also measured. After hospital discharge, the subjects were followed up via phone calls at one week, one month, and three months evaluating for pain at surgical incision sites as well as TPVB injections sites using NPS, amount of analgesics usage, potential adverse events such as nausea, emesis, itching, and infection.

3. *Statistical Analysis*

Data are presented as mean (SD), or median (interquartile range (IQR): 25th percentile - 75th percentile) for continuous variables, and frequency and percentage for categorical variables. The analysis of our primary outcome, the number of dermatomes at different follow-up time points, was performed by fitting a linear mixed model with the use of SAS Proc Mixed procedure, in which fixed effects including group (Ropi_Betamet_TPVB vs. Ropi_TPVB), time (0.25 h, 0.5 h, 2 h, 4 h, 8 h, 12 h, 24 h, 48 h, 72 h), time by group interaction term were adjusted as covariates in the mean model. An autoregressive covariance matrix was specified to account for correlations of repeated observations from the same individuals. The least square mean estimates and 95% confidence intervals for the outcome at different time points were calculated. The statistical comparisons of secondary outcomes were performed using the Wilcoxon rank sum test for continuous variables and Fisher’s exact test for categorical variables.

All the statistical analyses were performed using the statistical software SAS v9.4 (Cary, NC). A two-side p-value of less than 0.01 was considered to be statistically significant.

4. *Sample Size Considerations*

The sample size for this trial was determined for the primary outcome, TPVB duration measured by the number of dermatomes changes with time number of dermatomes changed over time, to assess the prolongation effects of betamethasone on TPVB. A sample size of 45 subjects per group (Ropi_Betamet_TPVB, Ropi_TPVB) was required to provide 80% power to detect a standardized Cohen’s d effect size of 0.7SD between the two TPVB groups with a significance level (alpha) of 0.01 using a two-sided two-sample unequal-variance t-test. To account for ~10% attrition rate due to loss of follow-up, we enrolled 50 subjects for both the Ropi_Betamet_TPVB and the Ropi_TPVB groups. An equal number of 50 subjects were enrolled for the control group, which would provide 80% power with an alpha level of 0.01 to detect Cohen’s d of 0.7SD for a continuous secondary outcome using two-sample unequal-variance t-test or detect an effect size (Cohen’s W) of 0.56 for a binary outcome using a 2 degrees of freedom Chi-Square test. Thus, in total, a sample size of n = 150 (50 per group) was selected.

## Results

**Figure 1:**
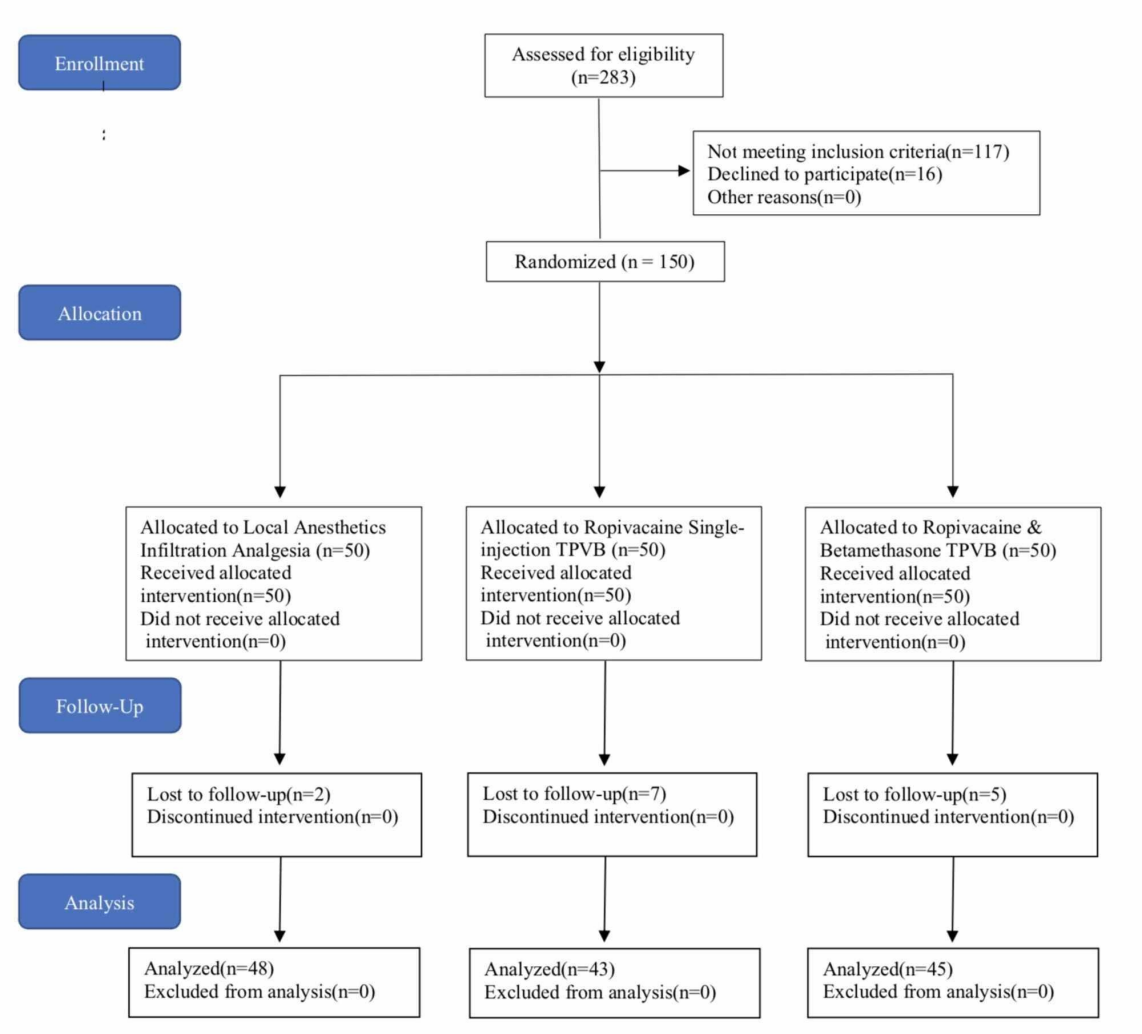
Flow Diagram in CONSORT Format. TPVB: Thoracic paravertebral block

A flow chart illustrates the process of this study (Figure 1).

A total of 283 patients were assessed for eligibility, 117 were excluded, 16 declined to participate and 150 entered randomization. Originally 50 patients were assigned to each group, due to loss of follow-up after patients were discharged, a total of 136 patients completed this study. In the control group, 48 patients received local infiltration through randomization per protocol, in the Ropi_TPVB group, 43 patients received TPVB with plain ropivacaine, and in the Ropi_Betamet_TPVB group, 45 patients received TPVB with ropivacaine mixed with betamethasone. There was no statistically significant difference among the groups for age, gender, weight and ASA status (Table 1).

**Table 1:** Patient Demographics and Baseline Characteristics. ASA: American Society of Anesthesiologists; TPVB: Thoracic paravertebral block.

Characteristics	Local infiltration (N = 48)	Ropi_TPVB (N = 43)	Ropi_Betamet_TPVB (N = 45)
Age, yr	48.19 (12.63)	51.79 (11.40)	47.98 (14.47)
Weight, kg	61.40 (9.47)	62.00 (10.23)	60.67 (12.00)
Gender			
Female	26 (054.17%)	26 (060.47%)	28 (062.22%)
Male	22 (045.83%)	17 (039.53%)	17 (037.78%)
ASA status			
1	34 (070.83%)	28 (065.12%)	29 (064.44%)
2	14 (029.17%)	15 (034.88%)	16 (035.56%)
Serum glucose preop (mmol/L)	5.13 (0.52)	5.17 (0.50)	5.07 (0.42)

Primary outcome

Betamethasone in TPVB resulted in longer blockade duration.

**Figure 2:**
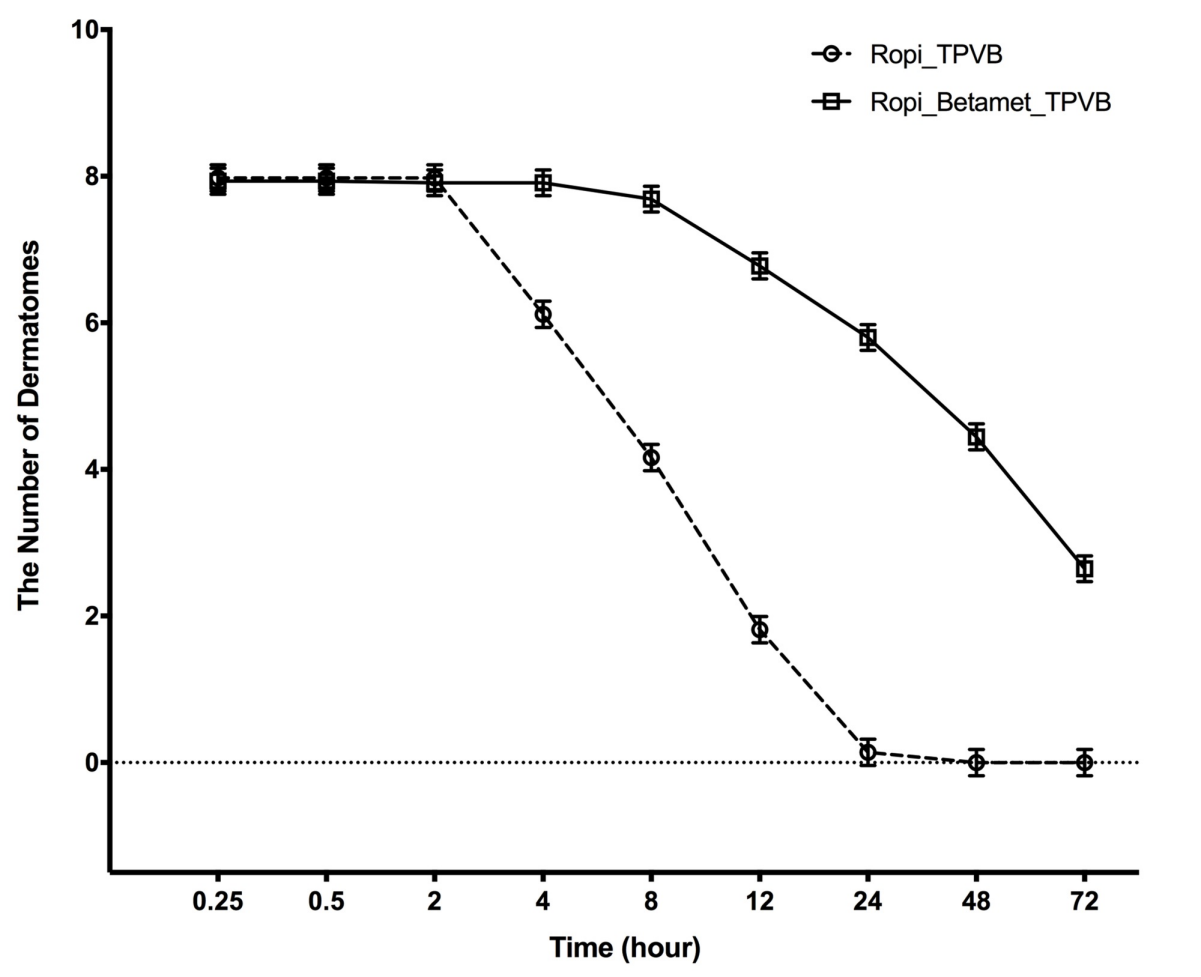
Chronological Changes in TPVB Dermatome Numbers. TPVB: Thoracic paravertebral block

We chose LC patients for this study as LC has the highest volume in this study center, but postoperative opioid usage in this procedure is not high in general, therefore we used regression of dermatome as a surrogate marker for pain control and analgesic usage (Figure 2).

There was no significant difference on the block onset of TPVB with or without betamethasone as both groups achieved approximately nearly eight levels of dermatomal coverage by 0.25 h. Block duration assessment was initiated when patients left the recovery room. There was no significant difference among groups on the interval between block placement and the start of block duration assessment, which included the surgical duration and recovery room time. As shown in Table 2, the number of dermatomes was significantly higher in the Ropi_Betamet_TPVB group as compared to the Ropi_TPVB group between 4 h and 72 h (p < 0.001). At 24 h, Ropi_TPVB patients had an average value of 0.14 (0.09) for the dermatomal numbers of blockade, while the Ropi_Betamet_TPVB patients on average still had a value of 5.80 (0.09) (Table 2). The group difference of 5.66 (95% CI: 5.38, 5.94) was statistically significant (p < 0.001). By the time of 48 h, Ropi_TPVB patients had no residual blocks yet Ropi_Betamet_TPVB patients still had an average of 4.44 dermatomes of blockade (difference = 4.44, 95% CI: 4.19, 4.70, p < 0.001). Of note, starting at 4 h until 72 h post block placement, the dermatomal numbers being blocked between the two TPVB groups all indicated statistically significant differences (P < 0.001).

**Table 2:** Summary of TPVB Dermatome Numbers with Time. *: Data are presented as least-square mean (standard error). TPVB: Thoracic paravertebral block

Outcome: # of dermatomes at:	Ropi_TPVB^*^ (N = 43)	Ropi_Betamet_TPVB^*^ (N = 45)	Difference estimate (95% CI)	P-value
0.25 h	7.98 (0.09)	7.93 (0.09)	-0.04 (-0.30, 0.21)	0.74
0.5 h	7.98 (0.09)	7.93 (0.09)	-0.04 (-0.30, 0.21)	0.74
2 h	7.98 (0.09)	7.91 (0.09)	-0.07 (-0.32, 0.19)	0.61
4 h	6.12 (0.09)	7.91 (0.09)	1.79 (1.54, 2.05)	<0.001
8 h	4.16 (0.09)	7.69 (0.09)	3.53 (3.27, 3.78)	<0.001
12 h	1.81 (0.09)	6.78 (0.09)	4.96 (4.71, 5.22)	<0.001
24 h	0.14 (0.09)	5.80 (0.09)	5.66 (5.40, 5.92)	<0.001
48 h	0 (0.09)	4.44 (0.09)	4.44 (4.19, 4.70)	<0.001
72 h	0 (0.09)	2.64 (0.09)	2.64 (2.39, 2.90)	<0.001

Secondary outcomes

1. Postoperative pain scores in TPVB patients with or without betamethasone were lower as compared to surgical port site infiltration control patients.

Shown in Table 3, patients of the two TPVB groups had statistically lower pain scores than patients in surgical infiltration group at all times up to 24 h (p < 0.001). The Ropi_Betamet_TPVB group had significantly lower pain scores than the Ropi_TPVB group at time points 8 h up to discharge at 72 h. The above differences in patient pain sensation were consistent with the TPVB dermatomal assessment shown in Figure 2, where there were significant differences of blockade effects between the Ropi_Betamet_TPVB group and Ropi_TPVB group. There was no incidence of persistent postsurgical pain in any group at one week, one month, and three months postoperatively.

**Table 3:** Secondary Outcomes and Adverse Events. Data are presented as median (IQR) or n (%). P-value from Wilcoxon rank sum tes­­­­­­t or Fisher’s exact test was presented. *: Frequency of analgesics 72 h: each time a rescue analgesic was given, it was counted as once.

					P-value	
Outcomes	Control (N = 48)	Ropi_TPVB (N = 43)	Ropi_Betamet_TPVB (N = 45)	Ropi_TPVB vs. Control	Ropi_Betamet_TPVB vs. Control	Ropi_Betamet_TPVB vs. Ropi_TPVB
Pain 0.5 h	1.0 (0.0 – 2.0)	0.0 (0.0 – 0.0)	0.0 (0.0 – 0.0)	<0.001	<0.001	0.13
Pain 2 h	2.0 (1.5 – 3.0)	0.0 (0.0 – 0.0)	0.0 (0.0 – 0.0)	<0.001	<0.001	0.06
Pain 4 h	3.0 (2.0 – 4.0)	0.0 (0.0 – 1.0)	0.0 (0.0 – 1.0)	<0.001	<0.001	0.44
Pain 8 h	3.0 (2.0 – 3.0)	2.0 (1.0 – 2.0)	0.0 (0.0 – 1.0)	<0.001	<0.001	<0.001
Pain 12 h	3.0 (2.0 – 4.0)	2.0 (2.0 – 3.0)	1.0 (0.0 – 1.0)	<0.001	<0.001	<0.001
Pain 24 h	2.0 (1.0 – 2.0)	1.0 (1.0 – 2.0)	0.0 (0.0 – 1.0)	0.001	<0.001	<0.001
Pain 48 h	1.0 (0.0 – 1.0)	1.0 (0.0 – 1.0)	0.0 (0.0 – 0.0)	0.018	<0.001	0.002
Pain 72 h	0.0 (0.0 – 0.5)	0.0 (0.0 – 1.0)	0.0 (0.0 – 0.0)	0.43	0.041	0.006
Pain at 1 week	0.0 (0.0 – 0.0)	0.0 (0.0 – 0.0)	0.0 (0.0 – 0.0)	1.00	1.00	1.00
Pain at 1 month	0.0 (0.0 – 0.0)	0.0 (0.0 – 0.0)	0.0 (0.0 – 0.0)	1.00	1.00	1.00
Pain at 3 months	0.0 (0.0 – 0.0)	0.0 (0.0 – 0.0)	0.0 (0.0 – 0.0)	1.00	1.00	1.00
Frequency of analgesics 72 h*	2.0 (2.0 – 3.0)	1.0 (0.0 – 1.0)	0.0 (0.0 – 0.0)	<0.001	<0.001	<0.001
Tramodol_mg_IV	0.0 (0.0 – 50.0)	0.0 (0.0 – 0.0)	0.0 (0.0 – 0.0)	0.05	<0.001	0.15
Parecoxib_mg_IV	80.0 (80.0 – 80.0)	0.0 (0.0 – 40.0)	0.0 (0.0 – 0.0)	<0.001	<0.001	0.008
Imrecoxib_mg_PO	0.0 (0.0 – 0.0)	0.0 (0.0 – 100.0)	0.0 (0.0 – 0.0)	0.08	0.034	<0.001
Dizziness, Yes	12 (025.00%)	05 (011.63%)	00 (000.00%)	0.10	<0.001	0.03
Nausea/Vomiting						
0	39 (081.25%)	37 (086.05%)	41 (091.11%)	1.00	0.53	0.42
1-3	09 (18.75%)	06 (13.95%)	04 (8.89%)			
Drowsiness, Yes	00 (000.00%)	00 (000.00%)	00 (000.00%)	1	1.00	1.00
Hypotension, Yes	00 (000.00%)	01 (002.33%)	00 (000.00%)	0.47	1.00	0.49
Respiratory depression, Yes	00 (000.00%)	00 (000.00%)	00 (000.00%)	1	1.00	1.00
Ileus, Yes	00 (000.00%)	00 (000.00%)	00 (000.00%)	1	1.00	1.00
Postop serum glucose (mmol/L) at 0.5 h	7.0 (6.5 – 7.8)	6.8 (6.2 – 7.9)	6.6 (6.0 – 7.3)	0.73	0.08	0.15
Delayed wound healing, Yes	00 (000.00%)	01 (002.33%)	00 (000.00%)	0.47	1.00	0.49
Wound infection, Yes	00 (000.00%)	00 (000.00%)	00 (000.00%)	1	1.00	1.00
First ambulation time, h	6.0 (3.0 – 8.0)	2.0 (1.0 – 2.0)	2.0 (1.0 – 2.0)	<0.001	<0.001	0.88
Length of stay, day	3.0 (3.0 – 3.0)	3.0 (3.0 – 3.0)	3.0 (3.0 – 3.0)	0.63	0.61	0.99

2. Postoperative analgesics consumption in patients with TPVB as compared to patients with surgical port site infiltration.

Shown in Table 3, the frequency of analgesics requested/administrated was significantly higher in the surgical infiltration group (control) as compared to both Ropi_TPVB group and Ropi_Betamet_TPVB group (P < 0.001). The administration frequency of supplemental analgesics in Ropi_Betamet_TPVB group was also significantly lower than that of the Ropi_TPVB group (P < 0.001). The intravenous opioid Tramadol consumption in the Ropi_Betamet_TPVB group was significantly different than that in the control group (P < 0.001). However, no significant difference was found between the Ropi_TPVB group and the control group, and between the Ropi_TPVB group and the Ropi_Betamet_TPVB group, respectively. The amount of IV Parecoxib in both TPVB groups was statistically lower than control, P < 0.001. In addition, the usage of IV Parecoxib in the Ropi_Betamet_TPVB group was statistically lower than that of Ropi_TPVB group, P = 0.008.

3. Perioperative functional outcomes and adverse events between surgical infiltration and TPVB groups.

Postoperatively, the median (IQR) time of each patient’s first-time ambulation in the control group, surgical infiltration only, was 6.0 (3.0-8.0) h, which was significantly greater (p < 0.001) than those in Ropi_Betamet_TPVB (2 (1.0-2.0) h), and Ropi_TPVB (2 (1.0-2.0) h) (Table 3). No dizziness was reported in the Ropi_Betamet_TPVB group, while the rates of it in the Ropi_TPVB and the control group were 11.63% and 25%, respectively. No significant difference in nausea/vomiting was identified among three groups, likely due to the usage of both intravenous and oral NSAIDs prior to opioids. There was one isolated delayed wound healing, and one reported hypotension in the Ropi_TPVB group. There was no incidence of wound infection, drowsiness, respiratory depression or ileus in any group.

## Discussion

Although there are several studies on the regression of spinal and epidural anesthesia, there are limited, if any, studies in TPVB in the current literature [3, 5]. To the best of our knowledge, this is the first RCT to systemically evaluate the waning course of sensory TPVB. Our study of dermatomes using cold sensation demonstrated the regressions of A 𝛿 fiber blockade after TPVB, which correlated roughly with the trend of somatic and visceral analgesia diminishing.

The salutary effects and safety of glucocorticoids in analgesia are well established [6-9]. However, controversy continues as to if perineural glucocorticoids have similar or better analgesic effects when compared to intravenous glucocorticoids. While some studies showed no difference [10, 11], others suggested perineural glucocorticoids have lower minimum dose and/or provide more effective analgesia at same dose than its intravenous counterpart [7, 12]. Hydrophilic/non-particulate glucocorticoid dexamethasone is most commonly used with the prolongation effect on peripheral nerve blockade for 6-8 h [13]. The search for longer acting local anesthetics or local anesthetic adjuvants with longer duration continues. Lipophilic/particulate glucocorticoids exhibit similar effectiveness and safety outside of intrathecal space as non-particulate counterparts [9, 14, 15] but with longer duration [16, 17]. Thus far particulate glucocorticoids in peripheral nerve blocks were only described in isolated studies [18-20]. The safety data for perineural glucocorticoids is lacking in general. Nonetheless, reported adverse events for particulate glucocorticoids were mostly related to intrathecal injection or intravascular injection, and no complications have been reported for peripheral nerve blocks [18, 20-23].

We chose to use betamethasone sodium phosphate/dipropionate combination for several reasons [1]. Betamethasone lacks mineral-corticoid activities and is of relatively smaller molecular size as compared to other glucocorticoids [2]. This combination of betamethasone is FDA approved, albeit for other indications than peripheral nerve block [3]. This combination of betamethasone has been used successfully in other peripheral nerve blocks such as great occipital nerve block [24], which was endorsed by the American Headache Society [4, 22]. This combination of betamethasone together with ropivacaine has been used safely in brachial plexus interscalene block [5,20]. When betamethasone dipropionate was applied directly to the transected tibial nerve in a rat model, it promoted nerve regeneration rather than induced nerve injury, which is a common concern for perineural adjuvant administration [6,25]. Pharmacokinetics studies of intramuscular injection betamethasone phosphate/betamethasone dipropionate showed that the active metabolite of betamethasone dipropionate has a half-life of approximately three days [26]. To minimize systemic effects, we used half of the above intramuscular dose of betamethasone through the route of perineural administration.

When it comes to glucocorticoid activity, we used in this study the dose of 2.5 mg of slow release betamethasone dipropionate phosphate and 1 mg immediate action betamethasone sodium phosphate, which adds up to about 3.125 mg dexamethasone in slow release format and 1.42 mg of dexamethasone in immediate release format, with a total activity of approximately 4.5 mg of dexamethasone, a common and arguably the optimal perineural dosing for dexamethasone as a local anesthetic adjuvant. Consistent with the small dose of betamethasone adopted in this study, we did not find any evidence of hyperglycemia, delayed wound healing or wound infection. We did not monitor serum glucose beyond the routine as recent multi-analysis of randomized control studies has shown a single dose perioperative dexamethasone has negligible effect on hyperglycemia, delayed wound healing or wound infection in non-diabetic and diabetic patients alike. Nonetheless, the lack of difference in serum glucose levels and wound infections is likely underpowered in the present study, future research may be directed to achieve the lowest possible dose of glucocorticoid until desired endpoints are met. Our study excluded diabetic patients, and it would be an interesting study when various types of diabetes, such as DM I, poorly controlled DM II, and well-controlled DM II, are involved.

In our study, the ultrasound-guided TPVB spread was approximately eight dermatomes by cold sensation, close to what was obtained by Cheema et al. [27], and was wider than what Cheema or Boezaart reported with radio‐opaque dye or methylene blue, respectively [28, 29]. This could be due to the assessment technique (direct visualization of dye spread vs. cold sensation), block technique (ultrasound vs. landmark), and the relatively low BMI in our study patient.

One limitation of this study is blinding. The performance of TPVB is not without any risks and we did not feel it ethically justified to perform sham TPVB in control patients, therefore patients are not blinded in terms of if they received TPVB. Secondly, the assessment of block onset was not blinded, as the single investigator (LL) who prepared all the medications, performed all the TPVB procedures, also performed the very first TPVB block level assessment at 0.25 h. However, all subsequent assessments were exclusively performed by a single blinded practitioner (XZ) after patients had left the recovery room. Another limitation of this study was the choice of LC procedure, which is common, has documented risk of persistent postsurgical pain, but typically not associated with a long length of hospital stay or significant disease burden. As a result, the assessments for TPVB duration were prematurely stopped at the time of discharge at 72 h. In addition, other than the duration of blockade, pain level assessment, and analgesics consumption, some important outcome measures such as patient satisfaction and quality of recovery were not sought. Future studies of TPVB with particulate glucocorticoids in more invasive procedures with longer than 72 h of stay are warranted. Even though the TPVB had significant impacts on perioperative pain scores, analgesics consumption, and functional status, there were no changes on the length of stays in this study, as the surgeons were not comfortable to send any LC patients home earlier than routine. As a result, the investigators did not feel the need to include cost of hospitalization parameter in this study.

A major strength of this study was its in-depth review of dermatomal subsiding pattern over time. We believe this to be a unique aspect to this study not previously published. Our research suggested that paravertebral administration of local anesthetic and low dose non-particulate and particulate glucocorticoids can attain substantial effects on postoperative analgesia on LC patients without significant side effects or complications. This study serves, in part, as a proof of concept for TPVBs as a reliable intervention for post-operative analgesia in abdominal surgery. It is the first RCT that demonstrated the superiority of unilateral TPVB over surgical local anesthetic infiltration for LC, also the first RCT that investigated the effectiveness and safety of particulate glucocorticoid adjuvants in TPVB for acute perioperative pain management.

## Conclusions

Our study is among the few that reported much longer than 24 h duration of single injection TPVB blockade achieved with a local anesthetic adjuvant resulting in improved perioperative analgesia and functional outcomes, and without significant adverse events.
